# Occupational Benzene Exposure and the Risk of Lymphoma Subtypes: A Meta-analysis of Cohort Studies Incorporating Three Study Quality Dimensions

**DOI:** 10.1289/ehp.1002318

**Published:** 2010-09-29

**Authors:** Jelle Vlaanderen, Qing Lan, Hans Kromhout, Nathaniel Rothman, Roel Vermeulen

**Affiliations:** 1 Institute for Risk Assessment Sciences, Utrecht University, Utrecht, the Netherlands; 2 Division of Cancer Epidemiology and Genetics, National Cancer Institute, Department of Health and Human Services, National Institutes of Health, Bethesda, Maryland, USA

**Keywords:** acute lymphocytic leukemia, benzene, chronic lymphocytic leukemia, Hodgkin lymphoma, leukemia, meta-analysis, multiple myeloma, non-Hodgkin lymphoma, occupational exposure

## Abstract

**Background:**

The use of occupational cohort studies to assess the association of benzene and lymphoma is complicated by problems with exposure misclassification, outcome classification, and low statistical power.

**Objective:**

We performed meta-analyses of occupational cohort studies for five different lymphoma categories: Hodgkin lymphoma (HL), non-Hodgkin lymphoma (NHL), multiple myeloma (MM), acute lymphocytic leukemia (ALL), and chronic lymphocytic leukemia (CLL).

**Data extraction:**

We assessed three study quality dimensions to evaluate the impact of study quality variations on meta-relative risks (mRRs): stratification by the year of start of follow-up, stratification by the strength of the reported acute myelogenous leukemia association, and stratification by the quality of benzene exposure assessment.

**Data synthesis:**

mRRs for MM, ALL, and CLL increased with increasing study quality, regardless of the study quality dimension. mRRs for NHL also increased with increasing study quality, although this effect was less pronounced. We observed no association between occupational benzene exposure and HL.

**Conclusions:**

Our meta-analysis provides support for an association between occupational benzene exposure and risk of MM, ALL, and CLL. The evidence for an association with NHL is less clear, but this is likely complicated by the etiologic heterogeneity of this group of diseases. Further consideration of the association between benzene and NHL will require delineation of risks by NHL subtype.

The International Agency for Research on Cancer (IARC) classified benzene as a group 1 carcinogen (carcinogenic to humans) in its 1982 and 1987 evaluations (IARC 1982, 1987) based primarily on reports of an association between occupational exposure to benzene and leukemia, particularly acute nonlymphocytic leukemia (ANLL), which consists primarily of acute myelogenous leukemia (AML). Recently, IARC updated its previous reviews of several chemicals and occupational exposure circumstances, including benzene, to reassess carcinogenicity and to consider potential associations with additional tumor sites (Baan et al. 2009). In that review, IARC determined for the first time that in addition to the confirmed association with ANLL, there was also limited evidence that benzene causes acute lymphocytic leukemia (ALL), chronic lymphocytic leukemia (CLL), non-Hodgkin lymphoma (NHL), and multiple myeloma (MM) in humans (Baan et al. 2009). At the same time, in recent years, there has been a plethora of reviews and meta-analyses of benzene and one or more lymphoid neoplasms, at times reaching diametrically opposed conclusions (Alexander and Wagner 2010; Bergsagel et al. 1999; Infante 2006; Kane and Newton 2010; Lamm et al. 2005; Savitz and Andrews 1997; Schnatter et al. 2005; Smith et al. 2007; Sonoda et al. 2001; Steinmaus et al. 2008; Wong and Fu 2005; Wong and Raabe 1995, 1997, 2000a, 2000b).

There are two fundamental challenges in using the large number of occupational cohort studies that have been published over the last 30 or so years when considering the relationship between occupational benzene exposure and the risk of lymphoid neoplasms. First, there have been substantial changes in testing procedures, diagnostic criteria, and categorization of lymphoid neoplasms over the last half-century (Aisenberg 2000; Harris et al. 2000; Linet et al. 2007; Morton et al. 2007), the time period in which follow-up of these occupational cohorts took place. Indeed, diagnostic criteria that were used in these cohort studies were based on a range of classification strategies, including the *International Classification of Diseases* (ICD), Revisions 7–9, and *ICD for Oncology*, Revision 3 (ICD-O3) (World Health Organization 1955, 1965, 1975, 2000). The changing views on the categorization of lymphoid neoplasms is illustrated by the current categorization of ALL and CLL as subtypes of NHL in the most recent World Health Organization disease classification (Swerdlow et al. 2008), although these entities have been reported separately from NHL in essentially all occupational cohort studies of benzene-exposed workers. Second, there is heterogeneity in occupational cohort studies with regard to industry, sample size, documentation and level of benzene exposure, and documentation of the percentage of a given cohort that had true, nontrivial exposure to benzene. Inadequate documentation, uncertain quality of follow-up, and, most problematic, potential inclusion of “unexposed” workers in “exposed” categories would have likely resulted in attenuation of the observed associations. Further, for the purpose of reviews or meta-analyses, it can be challenging to separate informative from potentially noninformative cohorts in the face of uncertain documentation of key epidemiological study design and exposure assessment characteristics.

Given the changing nature of the diagnosis of lymphoid neoplasms over time and the heterogeneity of occupational benzene cohort study quality in the literature, it is a challenge to discern the nature of the relationship between benzene and lymphoid neoplasms. To address this issue, we developed three strategies that we employ in a set of meta-analyses of occupational cohort studies for five lymphoma categories defined according to ICD-9: Hodgkin lymphoma (HL; ICD-9 code 201), NHL (ICD-9 200, 202), MM (ICD-9 203.0), ALL (ICD-9 204.0), and CLL (ICD-9 204.1).

We applied the first strategy to assess the potential impact of the gradual increase in the quality of hematologic diagnoses over the last decades. This strategy involved stratification of the studies in the meta-analyses based on the reported start of follow-up. We used the year 1970 as a cutoff point for stratification (approximate midpoint of follow-up of all studies included in this analysis). We based the second strategy on the established strong association between benzene and AML. We argue that any study that was not able to detect at least a suggestive association between benzene and AML most likely had serious methodologic limitations in one or more aspects of study design. Examples of possible limitations are trivial exposure to benzene in the studied cohort, inclusion of “unexposed” workers in “exposed” categories or flaws in the assessment (or categorization) of health effects (Goldstein and Shalat 2000). Therefore, we used the direction and significance level of a reported association between benzene and AML as proxies for the overall study quality (AML significance level).

We based the third strategy on the evaluation of the quality of the exposure assessment carried out in each cohort. High-quality exposure assessment is essential to discriminate exposed individuals from nonexposed individuals (Vlaanderen et al. 2008). We assigned an exposure assessment quality classification to each study based on an *a priori* defined classification scheme and used this classification as an additional proxy of study quality, reasoning that those cohort studies with the highest quality exposure assessment had the greatest ability to identify and include workers who were truly exposed to benzene in their analyses.

We hypothesized that application of the three study quality dimensions—stratification based on the start of follow-up, AML significance level, and exposure assessment quality—would identify a subgroup of occupational cohort studies that is most informative for the evaluation of the possible association between benzene and lymphoid neoplasms.

## Materials and Methods

### Study identification and data extraction

We conducted a search of PubMed (http://www.ncbi.nlm.nih.gov/sites/entrez) using the key words “benzene” and “cohort” or “case–control.” We included publications in the meta-analysis if they were published in the peer-reviewed literature, reported results for any of the five lymphoma subtypes (HL, NHL, MM, ALL, and CLL), and were conducted in the occupational setting. We checked references in all identified publications for additional studies. When more than one paper was published on the same cohort, we chose the publication with the highest quality exposure assessment [e.g., in the Australian petroleum workers cohort for AML, we chose the nested case–control study that included an elaborated exposure assessment approach (Glass et al. 2003) over a more recent update on the full cohort that included no detailed benzene exposure assessment (Gun et al. 2006)]. When multiple publications with similar quality of exposure assessment were published on the same cohort, we chose the most recent update (with the longest follow-up time). In this meta-analysis, we pooled risk ratios, odds ratios (ORs), and standardized mortality ratios (SMRs). ORs and SMRs can be interpreted as reasonable approximations of the risk ratio when the disease is rare, and these measures have been pooled with risk ratios for meta-analyses before (McElvenny et al. 2004). We use the term “relative risk” (RR) to refer to the risk ratio, the OR, or the SMR. We extracted RRs based both on incidence and mortality. However, if a publication reported both, we chose incidence over mortality in the meta-analysis.

### Risk estimates

To allow the inclusion of studies without quantitative exposure assessment in our analysis, we used only RRs for “any occupational benzene exposure” versus “background benzene exposure” in the meta-analyses. If publications reported only RRs stratified for cumulative exposure and not for “any occupational benzene exposure” versus “background benzene exposure,” we pooled RRs by summing observed and expected cases for studies that reported SMRs (percentage of RRs: AML, 4.8%; HL, 3.7%; NHL, 3.0%; MM, 3.8%; CLL, 5.6%) or by conducting a within-study random-effects meta-analysis of the nonreference exposure groups for studies that reported RRs or ORs (percentage of RRs: AML, 14.3%; NHL, 3.0%; MM, 7.7%; ALL, 5.9%; CLL, 16.7%). If publications reported only observed and expected number of cases and no RRs, we calculated RRs and estimated associated confidence intervals (CIs) with mid-P exact (Rothman and Boice 1979) (percentage of RRs: AML, 4.8%; HL, 7.4%; ALL, 17.6%). For publications that reported no observed cases for any of the lymphoma subtypes, we calculated continuity corrected RRs (observed and expected number of cases plus one) and we estimated associated CIs with mid-P exact (percentage of RRs: ALL, 11.8%; HL, 11.1%). If studies reported zero for the lower CI, we imputed a value of 0.1 to allow estimation of the variance (percentage of RRs: ALL, 5.9%; MM, 3.8%).

### Three strategies for the assessment of study quality dimensions

We stratified by the year of start of follow-up based on the information provided in the included publications (follow-up started before 1970 vs. follow-up started in or after 1970). The median start of follow-up in the stratum with studies that started follow-up before 1970 was 1947, and the median start of follow-up in the stratum with studies that started follow-up in 1970 or after was 1973.

We assigned AML significance level to each publication based on a two-sided *p*-value of the *z*-score, which we estimated by dividing the reported log RR for AML by its standard error. Based on the calculated AML significance level, we assigned one of the following categories (A–E) to each publication: A, AML RR > 1, *p* < 0.1; B, AML RR > 1, 0.1 ≤ *p* < 0.2; C, AML RR > 1, *p* ≥ 0.2; D, AML RR ≤ 1; E, no AML RR reported.

We assigned quality of exposure assessment (A–D) to each publication as follows: A, in the publication explicit quantitative exposure estimates for benzene were reported; B, in the publication semiquantitative estimates of benzene exposure or quantitative estimates of exposures containing benzene (e.g., gasoline) were reported; C, in the publication some industrial hygiene sampling results to indicate that benzene exposure was present in the cohort that was studied were reported; D, the publication qualitatively indicated that benzene exposure was present in the cohort.

### Statistical analyses

We conducted random-effects meta-analyses to pool the RRs reported in the included publications. We used an α of 0.05 to assess whether meta-relative risks (mRRs) were significantly elevated. We conducted the first set of meta-analyses on the full set of studies stratified for the start of follow-up (follow-up started before 1970 vs. follow-up started in or after 1970). We compared mRRs by strata using a test of interaction as suggested by Altman and Bland (2003).

We applied the study quality dimensions of AML significance level and quality of exposure assessment in two series of meta-analyses. The initial analysis in each series included all studies regardless of quality. In each subsequent analysis, we excluded the group of studies with the lowest AML significance level or the lowest quality of exposure assessment.

We used Cochran’s *Q*-test to assess between study heterogeneity in all meta-analyses. A *p*-value < 0.1 was considered to be statistically significant evidence for between study heterogeneity. We used *I*^2^ to describe the percentage of total variation across studies that was due to heterogeneity rather than chance (Higgins et al. 2003). For analyses that displayed significant between study heterogeneity, we assessed the sensitivity of the outcome of the meta-analysis for individual studies by excluding studies one at the time (jackknife analysis). We assessed publication bias with Eggers graphical test (Egger et al. 1997). We performed all meta-analyses in Stata (version 11; StataCorp LP, College Station, TX, USA).

## Results

We identified 44 publications that provided an RR for at least one of the lymphoma subtype–specific meta-analyses. We did not extract data from three publications: one study with likely underascertainment of cancer deaths as the result of the inability to identify the type of cancer for a number of cancer deaths (Infante 2005; Sorahan et al. 2005); one study for which we could not estimate the RR variance [a nested case–control study that did not report CIs (Ott et al. 1989)]; and one study that reported proportionate mortality ratios, which tend to underestimate the RR (Thomas et al. 1982). Table 1 lists all publications that contributed to the meta-analyses, their (assigned) cohort name, the (assigned) name of the subcohort (if relevant), the literature reference, the type of industry in which the study was performed, the follow-up period, the lymphoma subtype for which the publication was included (if reported with ICD code and revision), an indicator of whether RRs were based on incidence or mortality, the assigned AML significance level, and the assigned quality of exposure assessment. The earliest included publication dates from 1983, and the most recent publication was from 2008. For two cohorts we used non-peer-reviewed publications to extract RRs for MM (and NHL) that were not reported in the peer-reviewed publications (Atkinson et al. 2001; Delzell E, Sathiakumar N, Cole P, Brill I, unpublished report). Both reports were based on the exact same methodology and follow-up time as reports of these cohorts that appeared in the peer-reviewed literature (Glass et al. 2003; Sathiakumar et al. 1995). We included an RR for MM from a study by Decoufle et al. (1983) based on additional information that was reported in the preamble to the final Occupational Safety and Health Administration (OSHA) benzene standard of 1987 (OSHA 1987). We extracted NHL RRs for two studies by Wong and colleagues (Wong 1987a; Wong et al. 1993) from Wong (1998), a letter that provided results from additional analyses for these studies. Finally, there might have been a slight (nonidentifiable) overlap in the cohorts studied by Wong (1987a, 1987b) and Collins et al. (2003).

Table 2 shows the mRR based on random-effect meta-analyses for all studies and stratified by start of follow-up for AML and the five lymphoma subtypes (i.e., HL, NHL, MM, ALL, and CLL). The overall mRRs (95% CIs) for AML and ALL were significantly increased [mRR = 1.68 (1.35–2.10) and mRR = 1.44 (1.03–2.02), respectively]. The overall mRR for MM and CLL were slightly but not significantly elevated, whereas the overall mRRs for HL and NHL were close to unity. Stratified analyses by start of follow-up showed higher RRs for AML, NHL, and CLL for studies with a follow-up starting in or after 1970 than for studies that started the follow-up before 1970 (*p* < 0.10). We observed no significant difference in mRR between the follow-up strata for HL, MM, and ALL. We observed significant between-study heterogeneity for AML, NHL, and CLL overall and in the studies with start of follow-up before 1970 [see Supplemental Material, Figure 1 (doi:10.1289/ehp.1002318)]. Exclusion of the most influential studies/RRs (based on the distance of the RR to the mRR and the weight of the study) resulted in mRRs that were essentially similar (data not shown).

Table 3 shows mRRs based on random-effects meta-analyses stratified by AML significance level for AML, HL, NHL, MM, ALL, and CLL. As could be expected, the lymphoma mRRs based on only the studies that reported an RR for AML (A–D) are largely similar to the mRRs based on all the studies (A–E). These studies therefore provide a relatively unbiased representation of the full set of studies. All outcomes except HL demonstrated an increase in mRRs with increasing AML significance level. However, the 95% CIs successively widened as a result of the reduced number of studies/RRs that were retained with each increase in AML significance level. The increase in mRR was most pronounced for MM and ALL, and somewhat weaker for NHL and CLL. In contrast, the mRR for HL dropped with increasing AML significance level. We observed significant between-study heterogeneity for NHL and CLL in the subset of studies with AML significance level A (*p* < 0.10) [see Supplemental Material, Figure 2 (doi:10.1289/ehp.1002318)]. Jackknife analysis eliminating one study at the time demonstrated that, in the NHL analysis of the studies with AML significance level A, the RRs from Divine and Hartman (2000) and Delzell et al. (unpublished report) had considerable impact on the between-study heterogeneity. Exclusion of both RRs from this analysis resulted in a slight decrease in the mRR (95% CI) from 1.16 (0.77–1.76) to 1.12 (0.77–1.61), with an *I*^2^ (an estimate of the percentage of total variation across studies that was due to heterogeneity rather than chance) of 22.8% (*p* = 0.27). In the CLL analysis of the studies with AML significance level A, the RRs provided by Divine and Hartman (2000) and Rushton and Romaniuk (1997) appeared to be primarily responsible for the observed between-study heterogeneity. Exclusion of both RRs from the meta-analysis resulted in a slight decrease in the mRR from 1.39 (0.65–2.96) to 1.26 (0.65–2.43), with an *I*^2^ of 0% (*p* = 0.94).

Table 4 shows mRRs based on random-effects meta-analyses and stratified by quality of exposure assessment. mRRs for NHL, MM, and CLL increased with increasing quality of exposure assessment. The increase in mRR was most pronounced for MM and CLL. Forest plots for AML and the five lymphoma subtypes for all studies with quality of exposure categories A and B (A, quantitative exposure estimates for benzene; B, semiquantitative estimates of benzene exposure or quantitative estimates of exposures containing benzene) are shown in the Supplemental Material, Figure 3 (doi:10.1289/ehp.1002318). Jackknife analysis eliminating one study at the time demonstrated that in the set of studies with quality of exposure categories A and B, the RRs provided by Wong et al. (1993) (land-based cohort) and Rushton and Romaniuk (1997) had considerable impact on the observed between-study heterogeneity in the CLL analysis. Exclusion of both RRs from the meta-analysis resulted in a slight decrease in the mRR (95% CI) from 1.54 (0.72–3.31) to 1.46 (0.79–2.72), with an *I*^2^ of 0% (*p* = 0.43). The RR provided by Wong (1998) (gasoline distribution employees) had a considerable impact on the observed between study heterogeneity in the NHL analysis of the set of studies in quality of exposure categories A and B. Exclusion of this RR resulted in a slight increase in the mRR from 1.04 (0.63–1.72) to 1.27 (0.90–1.79), with an *I*^2^ of 0% (*p* = 0.78).

Cross-stratification of AML significance level and quality of exposure assessment with the stratification based on the start of follow-up, although limited by a loss of statistical power, showed that mRR patterns with increasing AML significance level and quality of exposure assessment [see Supplemental Material, Tables 1, 2 (doi:10.1289/ehp.1002318)] were generally consistent with the patterns observed when meta-analyses were stratified by start of follow-up (Table 2).

Egger’s test revealed no significant evidence for publication bias in the data available for AML, HL, NHL, ALL, or CLL [see Supplemental Material, Figure 4 (doi:10.1289/ehp.1002318)]. We observed evidence for bias for MM (*p* = 0.03), but Egger’s test became nonsignificant after exclusion of all quality of exposure assessment studies in category D (*p* = 0.72).

## Discussion

We conducted a series of meta-analyses on occupational cohort studies to assess the possible association between benzene and lymphoid neoplasms. Using different dimensions of study quality, we report evidence for an association between occupational benzene exposure and lymphoma subtypes MM, ALL, and CLL. For these subtypes, mRRs increased with increasing study quality, regardless of the strategy that was used to assess study quality. mRRs for NHL also increased with increasing study quality, although this effect was less pronounced. We did not observe an association between occupational benzene exposure and HL. Importantly, with the exception of a chance finding, the increase in mRRs for NHL, MM, ALL, and CLL with increasing study quality most likely reflects an actual underlying association with at least some of these lymphoma subtypes.

Because we observed mRR patterns consistent with a possible association between benzene and all lymphoma subtypes except HL, we formally explored quantitative exposure–response relations for NHL, MM, ALL, and CLL, including all studies in quality of exposure assessment category A (studies with quantitative estimates of benzene exposure) based on flexible meta-regression analyses (Vlaanderen et al. 2009). The relatively limited number of studies in category A resulted in uncertain and unstable predictions of the exposure–response curve for NHL, MM, and CLL (data not shown). For ALL, only one study in quality of exposure assessment category A was available that precluded conducting a meta-regression for this lymphoma subtype. Therefore, possible dose–response associations can only be discussed informally on a study-by-study basis.

### Assessment of study quality dimensions

We developed three different quality dimensions that reflect the substantial changes in diagnosis and categorization of lymphoid neoplasms over the last half century and the heterogeneity in occupational cohort studies with regard to industry, sample size, and documentation of benzene exposure. The generally higher RRs in the strata with studies that started follow-up in or after 1970 is consistent with better quality of lymphoma diagnosis in more recent years. The higher RRs are particularly noteworthy given that overall benzene exposure was likely reduced in workplaces after 1970–1980. Another secular trend in the quality of cohort studies over time was the greater use of incidence rather than mortality as end point (e.g., 91% of cohorts reporting CLL RRs with start of follow-up before 1970 used mortality as the end point vs. 43% for studies with start of follow-up in 1970 or later). It is possible that for less aggressive subtypes (e.g., CLL), subjects that died from other causes did not have lymphoma coded on their death certificate (Linet et al. 2007). However, cross-stratification of results suggested that stratification by period of follow-up explained more of the observed heterogeneity than stratification by mortality/incidence (data not shown). Although it has been suggested that the RR for leukemia subtypes observed in occupational studies might decrease with prolonged follow-up time (Richardson 2008), we found only modest evidence for this phenomenon for lymphoma subtypes. Substitution of the most recent RRs with those of previous updates did not materially change the results (data not shown).

Because the association between benzene and AML is established, we argue that a well-conducted large epidemiologic study on benzene and hemato- and lymphopoietic cancers should find such an association. If at least some evidence of association is not found, one could argue that there must be known or unknown methodologic limitations in the study design. Such studies would by extension most likely be noninformative regarding the association between benzene and lymphoid neoplasms. Naturally, one should realize that a failure to find evidence for an association could also be the result of insufficient statistical power. However, in our meta-analyses we observed that the strong increase in mRRs for AML with increasing AML significance levels was generally paralleled by increasing mRRs in lymphoma subtypes. In other words, studies that reported higher (and more significant) RRs for AML generally also reported higher RRs for NHL, MM, ALL, and CLL.

The quality of exposure assessment has a large impact on the ability of an epidemiological study to identify modest increased RRs. The relevance of our quality of exposure assessment approach was illustrated with the strong increase in mRRs for AML with increasing quality of exposure assessment. This trend provides support for our assumption that studies that conducted a more detailed benzene exposure assessment likely provide higher overall quality of evidence for the potential association of benzene with adverse health outcomes. Although one would expect that the study quality indicators for AML significance level and quality of exposure assessment to be highly correlated, this is not necessarily the case. For instance, we did observe five studies in the lowest category quality of exposure assessment (D) that still reported a significant increased RR for AML, and we observed two studies from quality of exposure assessment category B in the set of studies that reported an AML RR below unity (AML significance level category D). Therefore, the two study quality dimensions should be seen as complementary.

### NHL

We observed a moderate increased RR of NHL with increasing study quality. However, neither the overall mRR nor any of the strata-specific mRRs reached formal statistical significance. Because our formal meta-regression did not result in robust dose–response associations, we qualitatively explored exposure–response relations within each exposure assessment quality category A publication that provided RRs for NHL. Of the six exposure assessment quality category A studies that reported RRs for NHL only one study reported a significant increased RR (*p* for trend < 0.02) with increasing cumulative exposure to benzene (Hayes et al. 1997). In contrast, in three of six publications the authors reported no clear trend of RRs for NHL with increasing cumulative exposure to benzene (Bloemen et al. 2004; Collins et al. 2003; Schnatter et al. 1996), whereas the remaining two publications did not report on the quantitative relation between NHL and cumulative exposure to benzene (Rinsky et al. 2002; Wong 1987a). In addition to these six studies, two publications that included MM in the definition of NHL did report on the quantitative relation of NHL plus MM and cumulative exposure to benzene (Glass et al. 2003; Wong 1987b). One of these studies reported an initial increase in RR with increasing exposure to benzene followed by a drop in RR in the upper cumulative exposure group (Wong 1987b), whereas the other study reported no association (Glass et al. 2003). We note, however, that a recent meta-analysis including both case–control and cohort studies reported a significant elevated mRR for NHL when we restricted the analyses to the higher exposure groups and corrected for the healthy worker (inclusion) effect (Steinmaus et al. 2008).

Overall, the epidemiologic evidence for the association between NHL and benzene is conflicting. This is illustrated by three recent meta-analyses that were based on largely the same data but reached a diametrically opposite conclusion on whether exposure to benzene is associated to NHL (Alexander and Wagner 2010; Kane and Newton 2010; Steinmaus et al. 2008). The inconsistency in findings is partly explained by study quality and failure to correct for biases but might also to a certain extent be explained by the etiologic heterogeneity within this group of diseases. If some NHL subtypes [e.g., diffuse large B-cell lymphoma (DLBCL) or follicular lymphoma (FL)] are associated with benzene, but others are not, any NHL RR will be attenuated because of the inclusion of non-benzene-associated NHL subtypes. This is even further complicated by the fact that the distribution of NHL subtypes may vary considerably from population to population, which could lead to significant variation in reported associations between potential risk factors and total NHL (Muller et al. 2005).

A series of recent population-based case–control studies provide evidence that the association between some genetic and environmental factors varies between major NHL subtypes such as DLBCL and FL (Lan et al. 2009; Morton et al. 2008; Rothman et al. 2006; Skibola et al. 2009, 2010). Another series of recent case–control studies that used relatively high-quality retrospective exposure assessment methods have provided evidence that this might also be true for the association between benzene and NHL subtypes (Cocco et al. 2010; Miligi et al. 2006; Wong et al. 2009). The studies by Cocco et al. (2010) and Wong et al. (2009) reported a stronger association with benzene with FL [OR = 1.6 (95% CI, 0.9–2.9) and OR = 7.00 (95% CI, 1.45–33.70), respectively] than for DLBCL [OR = 0.9 (95% CI, 0.6–1.4) and OR = 0.66 (95% CI, 0.31–1.42), respectively]. The study by Miligi et al. (2006) did not report an RR for FL (due to the limited number of cases) but reported an OR of 2.4 (95% CI, 1.3–4.5) for DLBCL.

### MM

Our analyses are supportive of an association of benzene exposure with MM. mRRs increased considerably and reached near statistical significance regardless of the study quality dimension used except for the analyses stratified by AML significance level, where formal statistical significance was reached for the two highest quality strata. Our results are similar (albeit that the point estimates of the mRRs are slightly lower) to the results from a meta-analysis by Infante (2006), in which slightly different inclusion criteria were applied [mRR = 2.13 (95% CI, 1.31–3.46)]. Further evidence for an association between exposure to benzene and MM have been provided by two recent population-based case–control studies that reported increased MM RRs with increasing exposure to benzene (Cocco et al. 2010; Seniori Costantini et al. 2008). We qualitatively explored the quantitative exposure–response relation between benzene and MM. Two of eight exposure assessment quality category A studies reported an increase in RR with increasing cumulative exposure (Collins et al. 2003; Rinsky et al. 2002); in two studies the authors reported no clear trend of RRs for MM with increasing cumulative exposure to benzene (Atkinson et al. 2001; Schnatter et al. 1996); and four studies did not report on the quantitative relation between cumulative exposure to benzene and MM (Bloemen et al. 2004; Swaen et al. 2005; Wong 1987b; Yin et al. 1996). Therefore, although the evidence for an association between “any occupational benzene exposure” versus “background benzene exposure” and the RR of MM appears to be consistent, the evidence for an exposure–response relation between benzene and MM is more ambiguous. This would be explained partly by the much larger statistical power that is required to conduct quantitative exposure–response analysis, often a complication for small-scale occupational cohort studies.

### ALL

The association between exposure to benzene and ALL is difficult to study because the disease is rare in adults (Faderl et al. 2010). It is therefore noteworthy that our analyses do strongly suggest increased RRs for ALL. We were able to identify only two population-based case–control studies that explored benzene–ALL associations in adults (Adegoke et al. 2003; Richardson et al. 1992). One case–control study reported a (nonsignificantly) increased RR for ALL with a suggestion of an exposure–response relation (Adegoke et al. 2003), whereas the other study did not observe any cases with ALL (Richardson et al. 1992). Together, the evidence from both cohort and case–control studies are strongly suggestive of a positive association between exposure to benzene and the RR of adult ALL.

### CLL

Our analyses suggest that exposure to benzene is associated with an increased RR for CLL. This is in line with results from four recent case–control studies that reported RRs ranging from 1.4 to 2.05 (Cocco et al. 2010; Miligi et al. 2006; Seniori Costantini et al. 2008; Wong et al. 2009). Two of these case–control studies reported an increase in RR with increasing benzene exposure (Cocco et al. 2010; Seniori Costantini et al. 2008). Of the cohort studies with quantitative exposure assessment, one study reported that the RR for the group with higher cumulative exposure was higher than the RR for the group with lower exposure (Glass et al. 2003). However, two cohort studies reported no association with cumulative exposure to benzene (Collins et al. 2003; Rushton and Romaniuk 1997), whereas one study did not report on the quantitative relation between cumulative exposure to benzene and CLL (Bloemen et al. 2004).

## Conclusion

In line with the recent IARC evaluation of the carcinogenicity of benzene, our meta-analyses provide evidence for the association of occupational benzene exposure to MM, ALL, and CLL (Baan et al. 2009). Although these findings are suggestive, it is important to realize that most analyses were based on data sets of limited size. The evidence for an association between benzene and NHL (as defined in ICD-9) is less convincing, but this could be explained by the heterogeneity in the association for particular subgroups of this disease or by not accounting for certain biases. We observed no association between benzene and HL. The discussion on the association between benzene and NHL will likely benefit from NHL subtype–specific analyses. Unfortunately, most current occupational cohort studies lack sufficient statistical power to perform such detailed analyses. Cohort studies with central pathology review and well-designed case–control studies using state-of-the-art retrospective exposure assessment methods will be needed to help evaluate the extent to which occupational benzene exposure is associated with specific subtypes of NHL.

Finally, our overall findings, taken together with the substantial experimental and molecular epidemiologic evidence that benzene exposure alters key components of the immune system relevant for lymphomagenesis (e.g., CD4^+^ T-cell level and CD4^+^ T-cell:CD8^+^ T-cell ratio) (Lan et al. 2004), provide support that benzene is likely to be causally related to one or more subtypes of lymphoma.

## Figures and Tables

**Table 1:** Overview of publications included in the meta-analyses.

Cohort	Subcohort	Reference	Industry	Follow-up period	Included for outcomes (ICD^a^)	ICD revision^a^,^b^	I/M^c^	AML significance level^d^	Exposure assessment quality^e^
Australian petroleum workers cohort		Atkinson et al. 2001^f^	Petroleum industry	1980–1998	MM (203)	9	I	C	A
Australian petroleum workers cohort		Glass et al. 2003	Petroleum industry	1980–1998	AML (205.0, 208.0), CLL (204.1)	9	I	C	A
Australian petroleum workers cohort		Gun et al. 2006	Petroleum industry	1981–1999	ALL (204.0), NHL (200–202)	9	I	C	D
Beaumont, Texas, petroleum refinery cohort		Wong et al. 2001a	Petroleum industry	1945–1996	ALL (204.0), AML (205.0), CLL (204.1), MM (203), NHL (200, 202), HL (*)	8	M	C	D
Canadian petroleum company cohort		Schnatter et al. 1996	Petroleum industry	1964–1983	MM (203), NHL (200, 202.0, 202.1, 202.2, 202.9)	8	M	E	A
Canadian petroleum company cohort		Lewis et al. 2003	Petroleum industry	1964–1994	ALL (204.0), AML (205.0), CLL (204.1), HL (201)	9	I	D	D
Caprolactam workers		Swaen et al. 2005	Chemical industry	1951–2001	MM (31), HL (29)	B	M	E	A
Conoco chemical plant cohort		Decoufle et al. 1983	Chemical industry	1947–1977	MM (*)	8	M	E	D
Dow cohort		Bloemen et al. 2004	Chemical industry	1940–1996	AML (205.0, 206.0, 207.0), CLL (204.1), MM (203), NHL (200.0–200.8, 202.0, 202.8), HL (201)	9	M	C	A
Exxon cohort	Louisiana	Huebner et al. 2004	Petroleum industry	1970–1997	ALL (204.0), AML (205.0, 206.0, 207.0, 207.2), CLL (204.1), MM (203.0), NHL (200.0–200.2, 200.8, 202.0–202.2, 202.8–202.9), HL (201)	9	M	B	D
Exxon cohort	Texas	Huebner et al. 2004	Petroleum industry	1970–1997	ALL (204.0), AML (205.0, 206.0, 207.0, 207.2), CLL (204.1), MM (203.0), NHL (200.0–200.2, 200.8, 202.0–202.2, 202.8–202.9) HL (201)	9	M	A	D
Finnish oil refinery workers		Pukkala 1998	Petroleum industry	1967–1994	NHL (*), HL (*)	(*)	I	E	D
French gas and electric utility workers		Guenel et al. 2002	Gas and electric utility industry	1978–1989	ALL (*), AML (*), CLL (*)	O	I	D	B
Italian oil refinery		Consonni et al. 1999	Petroleum industry	1949–1991	NHL (200, 202), HL (201)	8	M	E	D
Martinez and Wilmington refinery and petrochemical plants, California		Tsai et al. 1993	Petroleum industry	1973–1989	NHL (200), HL (201)	8	M	E	D
Monsanto cohort		Collins et al. 2003	Chemical industry	1940–1999	AML (205.0, 206.0), CLL (204.1), MM (203), NHL (200, 202), HL (201)	8	M	A	A
NCI-CAPM		Yin et al. 1996	Multiple industries	1972–1987	ALL (*), MM (*)	9	I	A	A
NCI-CAPM		Hayes et al. 1997	Multiple industries	1972–1987	AML (205.0, 206.0, 207.0), NHL (200, 202)	9	I	A	A
Norway upstream petroleum industry		Kirkeleit et al. 2008	Petroleum industry	1981–2003	ALL (*), AML (*), CLL (*), MM (*), NHL (*), HL (*)	7	I	A	D
Paulsboro, New Jersey, refinery (Mobil)		Collingwood et al. 1996	Petroleum industry	1946–1987	NHL (200), HL (201)	8	M	E	D
Petrochemical workers, Texas City, Texas		Waxweiler et al. 1983	Petroleum industry	1941–1977	NHL (200), HL (201)	7	M	E	D
Petroleum manufacturing plant, Illinois, USA (Shell)		McCraw et al. 1985	Petroleum industry	1973–1982	ALL (*), AML (*), CLL (*)	8	M	A	D
Petroleum manufacturing plant, Illinois, USA (Shell)		Honda et al. 1995	Petroleum industry	1940–1989	NHL (*), HL (*)	9	M	E	D
Pliofilm cohort		Wong 1995	Chemical industry	1940–1987	AML (C)	C	M	A	A
Pliofilm cohort		Rinsky et al. 2002	Chemical industry	1950–1996	NHL (C), MM (C)	C	M	E	A
Port Arthur, Texas, refinery workers		Satin et al. 1996	Petroleum industry	1937–1987	ALL (204.0), AML (205.), CLL (204.1), MM (203), NHL (200, 202), HL (201)	8	M	D	D
Cohort	Subcohort	Reference	Industry	Follow-up period	Included for outcomes (ICD^a^)	ICD revision^a^,^b^	I/M^c^	AML significance level^d^	Exposure assessment quality^e^
Richmond and El Segundo refineries		Dagg et al. 1992	Petroleum industry	1950–1986	NHL (200), HL (201)	8	M	E	D
Sample of U.S. refineries		Kaplan 1986	Petroleum industry	1972–1980	MM (*), HL (*)	(*)	M	E	D
Service station workers in Nordic countries		Lynge et al. 1997	Service station workers	1970–1990	AML (*), CLL (*), MM (203), NHL (200, 202), HL (201)	7	I	C	C
Shell Deer Park refinery		Tsai et al. 1996	Petroleum industry	1948–1989	NHL (200), HL (201)	8	M	E	D
Shell Louisiana refinery		Tsai et al. 2003	Petroleum industry	1973–1999	NHL (200), HL (201)	8	M	E	D
Shoe workers cohort	Italian cohort	Fu et al. 1996	Shoe workers	1950–1990	MM (203), NHL (200, 202)	9	M	E	D
Shoe workers cohort	U.K. cohort	Fu et al. 1996	Shoe workers	1939–1991	MM (203), NHL (200, 202)	9	M	E	D
Swedish seamen working on product or chemical tankers		Nilsson et al. 1998	Petroleum tanker workers	1971–1978	MM (203), NHL (200, 202), HL (201)	8	I	E	D
Texaco crude oil workers		Divine and Hartman 2000	Petroleum workers	1946–1994	ALL (*), AML (*), CLL (*), MM (*), NHL (*), HL (201)	8	M	A	D
Texaco mortality study		Divine et al. 1999a	Petroleum industry	1947–1993	HL (201)	8	M	C	D
Texaco mortality study		Divine et al. 1999b	Petroleum industry	1947–1993	ALL (*), AML (*), CLL (*), MM (*), NHL (*)	8	M	C	D
Torrance, California, petroleum refinery		Wong et al. 2001b	Petroleum industry	1959–1997	ALL (204.0), AML (205.0), CLL (204.1), MM (203), NHL (200, 202), HL (*)	8	M	D	D
U.K. oil distribution and oil refinery workers	Refinery	Rushton 1993	Petroleum industry	1950–1989	ALL	A	M	D	D
U.K. oil distribution and oil refinery workers	Distribution	Rushton 1993	Petroleum industry	1950–1989	ALL	A	M	C	D
U.K. oil distribution and oil refinery workers		Rushton and Romaniuk 1997	Petroleum industry	1950–1993	AML (*), CLL (*)	9	M	A	A
U.K. oil distribution and oil refinery workers	Refinery	Sorahan 2007	Petroleum industry	1951–2003	MM (203), NHL (200, 202), HL (201)	9	M	E	D
U.K. oil distribution and oil refinery workers	Distribution	Sorahan 2007	Petroleum industry	1951–2003	MM (203), NHL (200, 202), HL (201)	9	M	E	D
Union Oil Company cohort	Oil and gas division	Delzell et al. 1992^f^	Petroleum industry	1976–1990	MM (203), NHL (200, 202)	9	M	A	D
Union Oil Company cohort	Refining division	Delzell et al. 1992^f^	Petroleum industry	1976–1990	MM (203), NHL (200, 202)	9	M	E	D
Union Oil Company cohort	Oil and gas division	Sathiakumar et al. 1995	Petroleum industry	1976–1990	AML (*)	9	M	A	D
U.S. chemical workers		Wong 1987a	Chemical industry	1946–1977	HL (201)	8	M	E	A
U.S. chemical workers		Wong 1987b	Chemical industry	1946–1977	MM (203)	8	M	E	A
U.S. chemical workers	Wong 1998	Chemical industry	1946–1977	NHL (200, 202)	8	M	E	A	
U.S. gasoline distribution employees	Land based	Wong et al. 1993	Petroleum industry	1946–1986	ALL (*), AML (*), CLL (*)	8	M	B	B
U.S. gasoline distribution employees	Marine	Wong et al. 1993	Petroleum industry	1946–1986	ALL (*), AML (*), CLL (*)	8	M	D	B
U.S. gasoline distribution employees	Land based and marine	Wong 1998	Petroleum industry	1946–1986	NHL (200, 202)	8	M	C	B

NCI-CAPM, National Cancer Institute–Chinese Academy of Preventive Medicine.

a (*), ICD revision or specific ICD code was not reported.

b A, deaths were coded according to a system developed by Statistics Netherlands (CBS); B, deaths were coded according to National Institute for Occupational Safety and Health life-table analysis system death categories; C, deaths were coded according to the ICD in effect at time of death.

c I, incidence; M, mortality.

d A, AML RR > 1, *p* < 0.1; B, AML RR > 1, 0.1 ≤ *p* < 0.2; C, AML RR > 1, *p* ≥ 0.2; D, AML RR ≤ 1; E, AML RR not reported.

e A, quantitative exposure estimates for benzene; B, semiquantitative estimates of benzene exposure or quantitative estimates of exposures containing benzene; C, some industrial hygiene sampling results; D, qualitative indication that benzene exposure had occurred.

f Non-peer-reviewed publication.

**Table 2:** mRRs (95% CIs) for AML and five lymphoma subtypes in cohort studies of workers exposed to benzene: stratification by start of follow-up.

Lymphoma subtype	All studies			Start follow-up before 1970			Start follow-up in or after 1970			Test for difference by follow-up strata (*p*-value)^a^
	*n* studies	*n* exposed cases	mRR	*n* studies	*n* exposed cases	mRR (start follow-up before 1970)	*n* studies	*n* exposed cases	mRR (start follow-up 1970 and later)	
AML	21	217	1.68 (1.35–2.10)*	12	119	1.43 (1.07–1.92)*	9	98	2.08 (1.59–2.72)	0.06
HL	27	146	0.99 (0.83–1.19)	19	123	1.01 (0.83–1.23)	8	23	0.91 (0.59–1.40)	0.67
NHL^b^	33	647	1.00 (0.89–1.13)*	22	452	0.93 (0.81–1.06)*	11	195	1.21 (0.94–1.55)*	0.07
MM	26	284	1.12 (0.98–1.27)	16	204	1.07 (0.93–1.24)	10	80	1.26 (0.92–1.71)	0.35
ALL	17	47	1.44 (1.03–2.02)	10	30	1.30 (0.88–1.92)	7	17	1.92 (1.00–3.67)	0.31
CLL	18	111	1.14 (0.78–1.67)*	11	69	0.87 (0.50–1.50)*	7	42	1.63 (1.09–2.44)	0.07

a Test of interaction (Altman and Bland 2003).

b NHL or lymphosarcoma/reticulosarcoma (preferred NHL if the study reported both).

* Significant evidence for between study heterogeneity (*p* < 0.1).

**Table 3:** mRRs (95% CIs) for AML and five lymphoma subtypes in cohort studies of workers exposed to benzene: stratification by AML significance level.

Lymphoma subtype	AML significance level^a^	*n* studies	*n* exposed cases	mRR
AML	A–E (all studies)	21	217	1.68 (1.35–2.10)*
	A–D	21	217	1.68 (1.35–2.10)*
	A–C	16	192	1.88 (1.56–2.27)
	A–B	11	132	2.20 (1.77–2.72)
	A	9	108	2.48 (1.94–3.18)

HL	A–E (all studies)	27	146	0.99 (0.83–1.19)
	A–D	12	69	0.99 (0.77–1.27)
	A–C	9	39	0.82 (0.59–1.15)
	A–B	5	7	0.47 (0.22–0.99)
	A	4	7	0.50 (0.23–1.08)

NHL^b^	A–E (all studies)	33	647	1.00 (0.89–1.13)*
	A–D	15	383	0.97 (0.81–1.16)*
	A–C	13	344	0.99 (0.81–1.21)*
	A–B	7	130	1.21 (0.85–1.72)*
	A	6	101	1.16 (0.77–1.76)*

MM	A–E (all studies)	26	284	1.12 (0.98–1.27)
	A–D	14	160	1.15 (0.95–1.40)
	A–C	12	137	1.19 (0.94–1.49)
	A–B	7	69	1.49 (1.13–1.95)
	A	6	56	1.56 (1.11–2.21)

ALL	A–E (all studies)	17	47	1.44 (1.03–2.02)
	A–D	17	47	1.44 (1.03–2.02)
	A–C	11	29	1.41 (0.90–2.19)
	A–B	7	16	1.74 (0.90–3.36)
	A	5	12	1.74 (0.77–3.90)

CLL	A–E (all studies)	18	111	1.14 (0.78–1.67)*
	A–D	18	111	1.14 (0.78–1.67)*
	A–C	13	93	1.19 (0.74–1.90)*
	A–B	8	57	1.37 (0.73–2.56)*
	A	6	45	1.39 (0.65–2.96)*

a A, AML RR > 1, *p* < 0.1; B, AML RR > 1, 0.1 ≤ *p* < 0.2; C, AML RR > 1, *p* ≥ 0.2; D, AML RR ≤ 1; E, AML RR not reported.

b NHL or lymphosarcoma/reticulosarcoma (preferred NHL if the study reported both).

*Significant evidence for between study heterogeneity (*p* < 0.1).

**Table 4:** mRRs (95% CIs) for AML and five lymphoma subtypes in cohort studies of workers exposed to benzene: stratification by exposure assessment quality.

Lymphoma subtype	AML significance level^a^	*n* studies	*n* exposed cases	mRR
AML	A–D (all studies)	21	217	1.68 (1.35–2.10)*
	A–C	10	108	1.73 (1.26–2.38)
	A–B	9	95	1.82 (1.25–2.66)
	A	6	71	2.32 (1.55–3.47)

HL	A–D (all studies)	27	146	0.99 (0.83–1.19)
	A–C	5	16	0.99 (0.58–1.71)
	A–B	4	6	0.98 (0.36–2.67)
	A	4	6	0.98 (0.36–2.67)

NHL^b^	A–D (all studies)	33	647	1.00 (0.89–1.13)*
	A–C	8	106	1.03 (0.70–1.51)*
	A–B	7	69	1.04 (0.63–1.72)*
	A	6	50	1.27 (0.90–1.79)

MM	A–D (all studies)	26	284	1.12 (0.98–1.27)
	A–C	9	37	1.15 (0.74–1.79)
	A–B	8	28	1.48 (0.96–2.27)
	A	8	28	1.48 (0.96–2.27)

ALL	A–D (all studies)	17	47	1.44 (1.03–2.02)
	A–C	4	11	1.26 (0.5–3.16)
	A–B	4	11	1.26 (0.5–3.16)
	A	1	5	2.80 (0.27–29.23)

CLL	A–D (all studies)	18	111	1.14 (0.78–1.67)*
	A–C	8	61	1.38 (0.71–2.69)*
	A–B	7	53	1.54 (0.72–3.31)*
	A	4	43	2.44 (0.88–6.75)

a A, quantitative exposure estimates for benzene; B, semiquantitative estimates of benzene exposure or quantitative estimates of exposures containing benzene; C, some industrial hygiene sampling results; D, qualitative indication that benzene exposure had occurred.

b NHL or lymphosarcoma/reticulosarcoma (preferred NHL if the study reported both).

*Significant evidence for between study heterogeneity (*p* < 0.1).
